# MiRNAs in Hematopoiesis and Acute Lymphoblastic Leukemia

**DOI:** 10.3390/ijms24065436

**Published:** 2023-03-12

**Authors:** Diana Karen Mendiola-Soto, Diego Alberto Bárcenas-López, Carlos Jhovani Pérez-Amado, Gabriela Marisol Cruz-Miranda, Juan Manuel Mejía-Aranguré, Julian Ramírez-Bello, Alfredo Hidalgo-Miranda, Silvia Jiménez-Morales

**Affiliations:** 1 Laboratorio de Genómica del Cáncer, Instituto Nacional de Medicina Genómica, Mexico City 14610, Mexico; 2 Programa de Doctorado en Ciencias Biomédicas, Universidad Nacional Autónoma de México, Mexico City 04510, Mexico; 3 Programa de Doctorado en Ciencias Biológicas, Universidad Nacional Autónoma de México, Mexico City 04510, Mexico; 4 Programa de Maestría y Doctorado, Posgrado en Ciencias Bioquímicas, Universidad Nacional Autónoma de México, Mexico City 04510, Mexico; 5 Departamento de Endocrinología, Instituto Nacional de Cardiología Ignacio Chávez, Mexico City 14080, Mexico

**Keywords:** miRNAs, hematopoiesis, acute lymphoblastic leukemia, miRNA biomarkers, ALL subtypes

## Abstract

Acute lymphoblastic leukemia (ALL) is the most common kind of pediatric cancer. Although the cure rates in ALL have significantly increased in developed countries, still 15–20% of patients relapse, with even higher rates in developing countries. The role of non-coding RNA genes as microRNAs (miRNAs) has gained interest from researchers in regard to improving our knowledge of the molecular mechanisms underlying ALL development, as well as identifying biomarkers with clinical relevance. Despite the wide heterogeneity reveled in miRNA studies in ALL, consistent findings give us confidence that miRNAs could be useful to discriminate between leukemia linages, immunophenotypes, molecular groups, high-risk-for-relapse groups, and poor/good responders to chemotherapy. For instance, miR-125b has been associated with prognosis and chemoresistance in ALL, miR-21 has an oncogenic role in lymphoid malignancies, and the miR-181 family can act either as a oncomiR or tumor suppressor in several hematological malignancies. However, few of these studies have explored the molecular interplay between miRNAs and their targeted genes. This review aims to state the different ways in which miRNAs could be involved in ALL and their clinical implications.

## 1. Introduction

Acute lymphoblastic leukemia (ALL) is the most common cancer type in children under 15 years old [1]. This hematologic malignancy is characterized by a clonal expansion of immature lymphoid progenitor cells [2]. The current treatment for ALL is based on predicted risk of relapse, determined by age and white blood cell count (WBC) at diagnosis, infiltration to other organs, immunophenotype, and presence of cytogenetic and molecular alterations [3]. When comparing developed versus developing countries, mortality is 1–2% and 15–36.1%, 5-year disease-free survival is about 90% and 54.9%, and the proportion of patients with a high risk to relapse is 33% and 50%, respectively [4,5,6]. Relapsed ALL occurs in about 15–20%, but in children from low-income countries, it is up to 35%, which displays high rate of early age mortality [7,8,9].

One of the main goals is to bridge the gap between genotype and clinical prognosis; consequently, a great number of investigations have been developed to achieve a better understanding of each aspect of ALL. In this sense, it is known that epigenetic deregulation plays a key role in leukemogenesis, where non-coding RNAs (ncRNAs) have a pivotal role, acting as epigenetic regulators and enhancers and regulating intra- and inter-chromosomal interactions [10]. ncRNAs are broadly divided into two groups: housekeeping and regulatory (rncRNA). rncRNAs are synthesized at a specific phase of development or in response to some external stimuli and are classified according to their transcript size in small ncRNA (sncRNA) (<200 nucleotides) and long ncRNA (lncRNA) (>200 nucleotides). sncRNAs are very diverse, but micro-RNAs (miRNAs) are among the most studied [11,12,13]. MiRNAs were first described in 1993 by Lee et al., who identified *C. elegans* genes that control the timing of larval development, the lin-4. The authors discovered that lin-4 gene did not code for a protein, but instead produced a pair of sncRNAs [14]. To date, it is well-known that miRNAs play a relevant role in diverse biological processes and that their expression are disease and tissue specific [15]. Indeed, many efforts have been made to identify miRNA biomarkers in many human diseases.

## 2. MiRNAs’ Features and Biogenesis

**Figure 1:**
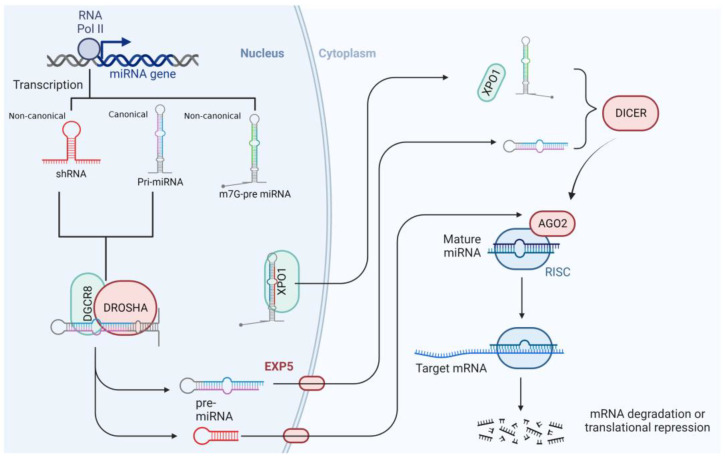
MiRNA biogenesis pathways. Schematic diagram of the canonical and non-canonical miRNA biogenesis process. In the canonical pathway, a primary miRNA transcript produced by RNA polymerase II is processed by the Drosha microprocessor in the nucleus. The generated pre-miRNA is transported to the cytoplasm in an EXP5-Ran GT-dependent manner and further processed by the Dicer microprocessor to generate a mature miRNA. The mature miRNA is loaded onto RISC to inhibit the translation of a target mRNA to conduct degradation. The non-canonical pathway is Drosha or Dicer dependent. AGO2, Argonaut protein 2; DGCR8, DiGeorge critical region 8; EXP5, exportin-5; RISC, RNA-induced silencing complex; XPO1, exportin-1.

MiRNAs are genomically encoded, untranslated RNA molecules that are, on average, 22 nucleotides in length [16]. Most miRNAs are generated through the canonical miRNA biogenesis pathway that is dependent on Drosha and Dicer; meanwhile, a few of them are processed by non-canonical miRNA pathways [17,18]. Many miRNAs are transcribed from DNA sequences into poly-adenylated and capped primary miRNAs (pri-miRNAs) molecules of >1 kilobase [16,19]. In the canonical miRNA biogenesis pathway, the pri-miRNA is endonucleolytically cleaved by the nuclear microprocessor complex formed by the RNase III enzyme Drosha and the DiGeorge critical region 8 (DGCR8) protein (also called Pasha) into a 60–70 nucleotides long precursor miRNA (pre-miRNA). After that, pre-miRNA can fold back on itself and form a hairpin loop structure that is transported out of the nucleus toward the cytoplasm by exportin-5 (EXP-5) in a complex with Ran-GTP [20]. Once in the cytoplasm, pre-miRNA is separated into two single-stranded molecules (guide and passenger strands), which, together with the RNA-induced silencing complex (RISC), form the RISC loading complex (RLC). This is a multi-protein complex that is composed by the RNase Dicer, the double-stranded RNA-binding domain protein of the transactivation response element RNA-binding protein (TRBP) and Protein kinase RNA (PKR) activator (PACT), and the core component Argonaut (Ago) proteins. Then the passenger strand is released, whereas the guide strand (the functional form) can act as a gene-expression regulator [21,22,23]. MiRNA biogenesis starts with the processing of RNA polymerase II/III transcripts post- or co-transcriptionally (Figure 1) [24].

About half of all identified miRNAs are intragenic and processed mostly from introns, and few of them are located within the exons of protein-coding genes. The remaining are intergenic, transcribed independently of a host gene by RNA polymerase II, and regulated by their promoters [22]. Sometimes miRNAs are transcribed as a family, meaning that they are part of one long transcript, called a cluster, with similar seed regions [23].

Studies have suggested that miRNAs are shuttled between different subcellular compartments to control the rate of translation and even transcription [21]. Most of them can regulate the gene expression by binding the 3′ untranslated regions (UTRs) of targeted mRNAs in an imperfectly complementary sequence manner. However, the interaction between miRNAs with 5′UTR, coding sequence, and gene-promoter regions has also been reported [25]. In addition to the role as transcription repressors, there is a phenomenon called RNA activation (RNAa) that was proven to be a sequence-dependent gene regulation mechanism evolutionarily conserved from *C. elegans* to mammals [26]. Synthetically designed double-strand RNAs capable of turning on gene expression are termed small/short activation RNAs (saRNAs). RNAa-mediated gene activation seems to depend on Ago protein and correlates with activating chromatin modification at the saRNA-targeted site. In terms of its targeting mechanism(s), saRNA can either act on promoter DNA or nascent transcripts, as there are works in the literature that support both cases [27,28,29].

## 3. MiRNAs and Hematopoiesis

Hematopoiesis is an active, continuous, and highly regulated process by which the production and repopulation of blood cells are carried out [30,31]. Hematopoiesis occurs in the bone marrow (BM), where hematopoietic stem cells (HSCs) with self-regenerative, multipotent, and highly proliferative capacity give rise to multipotent progenitors (MPPs) that can differentiate into the common myeloid (CMP) or lymphoid (CLP) progenitors [30,32]. The CMP differentiation process is called myelopoiesis; it allows for the production of erythrocytes, megakaryocytes/platelets, granulocytes (basophils, eosinophils, neutrophils), and monocytes/macrophages. CLP development, defined as lymphopoiesis, produces B- and T-lymphocytes (B-cells and T-cells, respectively) and natural killer (NK) cells [32]. The final fate of HSCs differentiation is highly regulated by the interaction between this kind of cells with surrounding cellular elements, genomic factors, and microenvironmental conditions that arise in response to hematopoietic demands under normal and pathological circumstances (infectious and inflammatory processes) [30,32,33,34,35]. MiRNAs are among the main modulators of the expression of genes involved in the regulation of cell-differentiation programs [32,36,37,38]. Gene expression analysis in purified hematopoietic populations has shown that specific miRNAs can regulate the differentiation and maturation stages of distinct hematopoietic cell lines [32,36,38,39,40]. For example, under normal conditions, it has been observed that miR-520h is highly expressed only in HSCs, while miR-129 expression is reduced in HSCs but increased in the granulocytic colony-forming unit (CFU-G), positively regulating granulocyte differentiation [32]. Additionally, miR-125 expression has been found to be increased in HSCs, thus indirectly promoting its expansion and compromising the differentiation toward myeloid lineage, particularly to granulocytes and macrophages [32,37,40,41].

**Figure 2:**
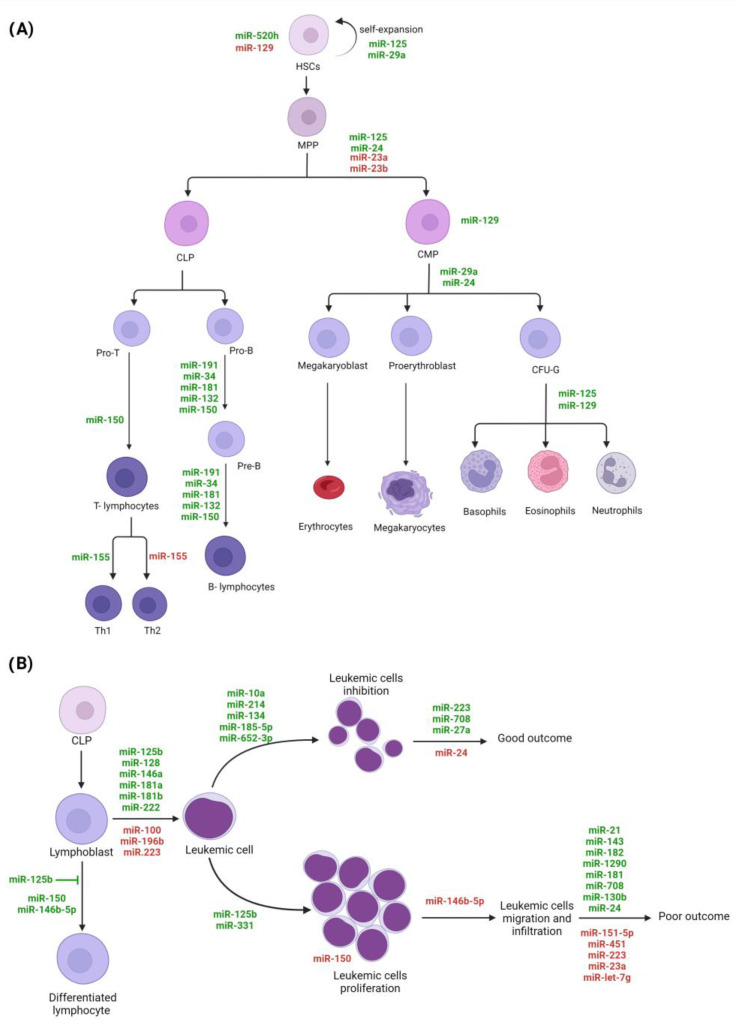
The miRNAs potentially implicated in hematopoiesis and leukemogenesis. (**A**) Hematopoiesis process is schematized, and examples of miRNAs expressed involved in the cell differentiation and maturation are indicated. (**B**) The miRNAs with dysregulated expression that are potentially involved in oncogenic lymphoblast transformation and progression are shown. MiRNAs in red means downregulation, and miRNAs in green means upregulation; HSCs, hematopoietic stem cells; MPP, multipotent progenitors; CLP, common lymphoid progenitors; CMP, common myeloid progenitors; CFU-G, granulocytic colony-forming unit.

Likewise, several miRNAs, such as miR-150, miR-155, miR-181, miR-34a, miR-132, miR-191, miR-24, and the miR-17-92 cluster, have been shown to be important regulators of lymphopoiesis [32,42,43]. Studies indicate that miR-191, miR-34a, miR-181, miR-132, and miR-150 modulate the pro-B- to pre-B-cell stage transition and B-cells’ maturation [32,42,43]. Some miRNAs have shown a tissue-specific effect; for example, while miR-150 expression in BM CLPs regulates pro-B-cell to pre-B-cell transition, in CLPs from thymus, miR-150 suppresses B lineage differentiation and increases the development of T cells (Figure 2A) [42]. Additionally, it has been observed that miRNAs modulate the differentiation of lymphocyte subpopulations; for instance, the presence of miR-155 induces the differentiation of T cells to Th1 subtype, while its absence promotes differentiation to the Th2 subtype [36,42,44,45].

Furthermore, it has also been observed that the alteration in the expression of miRNAs can change the fate of cell lineages [46,47]. For example, the deletion of miR-23a and miR-23b in HSCs correlates with a preferential differentiation toward myeloid lineage over B-cell differentiation [36,38]. The overexpression miR-29a contributes to the maintenance of an undifferentiated state and self-renewal capacities of HSCs and increased myelopoiesis; and miR-24 overexpression induces myelopoiesis instead lymphopoiesis (Figure 2A) [36,43].

Oncogenic miR-125b, a microRNA expressed during early T-cell development, enhances proliferation and blocks differentiation in TLX3+ T-ALL by repressing *ETS1* and *CBFβ* genes, critical transcription factors for T-lineage [48]. MiR-125b also participates in metabolic reprogramming in T-ALL, increasing glucose uptake and oxygen consumption in CD4-negative progenitor T cells [49]. MiR-150 exerts a tumor suppressor role in T-ALL by regulating several cell-cycle genes, and its expression is reduced in leukemic T-cells with active mTOR signaling [50]. MiR-146b-5p is another tumor suppressor whose expression is negatively regulated by transcription factor *TAL1*, leading to enhanced migration and invasion in vitro and lower overall survival in mouse models (Figure 2B) [51].

Due to the central role of miRNAs as regulators of many genes, alterations in these transcripts (mutations, polymorphisms, and expression changes) can modify hematopoietic development and manifest malignant phenotypes [46,47,52,53]. MiRNAs can also regulate genes involved in signaling pathways, such as the Notch, MAPK, and PI3K/AKT pathways, that are common amongst many types of leukemias [54]. For instance, it has been reported that the overexpression of miR-125b and miR-331 is associated with myeloid leukemia and ALL, respectively [36,55]. In addition, it has been observed that the ectopic expression of let-7c promotes granulocytic differentiation in AML [40], the low expression of miR-22 modifies the differentiation of monocytes in normal and leukemic states [40], and changes in the miR-17-92 cluster expression have resulted in the appearance of lymphoproliferative disorders [42]. A recent study identified miRNAs such as miR-125b-5p, miR-155-5p, miR-181a-5p, and miR-19a-3p and coding genes such as *BCL2*, *TP53*, *KIT*, and *MYB* with the highest number of interactions in several forms of leukemia and other hematological malignancies, including chronic lymphocytic leukemia (CLL) and myelodysplastic syndromes [56]. 

## 4. MiRNAs’ Role in Acute Lymphoblastic Leukemia 

**Figure 3:**
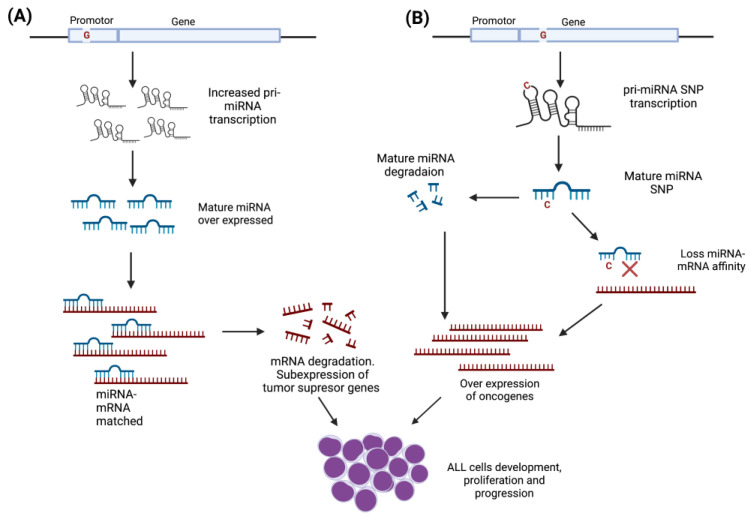
SNPs’ functional effect on miRNAs’ activity. Gene localization of SNPs will determine the effect on miRNA activity. (**A**) SNPs located in the promoter region could modify the expression levels of the mature miRNA, altering the interaction with mRNA target that codes for transcriptional factors involved in the development and differentiation of lymphoid blasts. The interaction between miRNA–mRNA target could promote the subexpression of tumor-suppressor genes, leading to the development, proliferation, and progression of leukemic cells. (**B**) SNPs located on the gene structure would give rise to unstable miRNAs that increase their degradation rate or whose sequence change prevents the miRNA–mRNA target interaction, promoting the overexpression of oncogenes and eventually the progression of leukemic cells.

miRNAs have the potential to affect the expression of a large number of genes involved in the origin and evolution of pediatric ALL. The abnormal function of miRNA expressions is one of the main epigenetic mechanisms that plays a relevant role in the development of ALL and other leukemia types [57,58,59]. Mutations and single nucleotide polymorphisms (SNPs) are among the principal factors that can modify miRNAs’ activity (Figure 3), and, in addition to their aberrant expression, they support ALL progression and might modulate the treatment response [60,61,62,63].

### 4.1. MiRNAs Polymorphisms as Risk Factors

It is well-known that SNPs located in miRNAs could alter the interaction miRNA-mRNA target or reduce the miRNA gene stability and, hence, its half lifetime. Even though their molecular mechanisms in the ALL pathogenesis is not well understood, several SNPs located in miR-146a, miR-196a-2, miR-499a, and miR-612 have been associated with ALL risk.

MiR-146a. An rs2910164G/C placed in miR-146a has been described as a functional SNP that causes a G:U pair to C:U mismatch in the stem structure of miR-146a precursor, which affects its binding specificity to its targets [64]. The rs2910164G/C was first associated with ALL by Hasani et. al., who reported that the C allele increases the risk for ALL in the Iranian population [65]. Furthermore, Zou et. al. found that the rs2910164 CC genotype increased the ALL risk in children from Asia [63]. Recently, an association in a gender-dependent manner among rs2910164G/C and ALL in Mexican children was published. The rs2910164C allele was identified as a risk factor to ALL in males [66]. However, controversial results have been found in Taiwan by Pei et. al., who documented that miR-146a rs2910164G allele is a protective marker for childhood ALL [62]. Studies in Thai, Indian, and Chinese populations did not observe an association between rs2910164G/C and ALL [61,67,68]. The study performed in patients from India also included rs57095329A/G in miR-146a, but no association with ALL was detected [61].

MiR-196a. The rs11614913 C/T polymorphism, which lies in the mature sequence of miR-196a, negatively impacts endogenous processing of pre-miRNA and its mature form; it has also been associated with various malignancies [69,70,71]. The CC genotype of this variant increases the expression levels of mature miR-196a2 and affects the binding of mature miR-196a2 to its target mRNA when compared with the rs11614913TT genotype [72]. The TC genotype, as well as CC/TC compared with the TT genotype of this variant, was found to be associated with a significantly increased risk for ALL susceptibility in Chinese children [73]. Then, in 2017, these results were replicated by Rakmanee et. al. in the Thai population, and they also reported an association of heterozygote TC genotype, both alone and in combination with CC genotype (TC + CC), increasing the risk of ALL in pediatric patients [74]. In addition, in Mexican cases, it was observed that the miR-196a rs11614913T allele confers risk to ALL in females [66]. Conversely, another study from Taiwan showed no association of the rs11614913C/T variant with ALL, thus highlighting the lack of reproducibility even in studies with populations with a similar genetic background [75].

MiR-499. The miR-499 rs3746444 involves an A/G nucleotide substitution which leads to a change from A:U pair to G:U mismatch in the stem structure of pre-miR-499 [65]. To date, several case-control studies have demonstrated that this variant is implicated in a variety of cancers [76,77,78,79,80]. The rs3746444G allele was found to be associated with a lower risk for ALL in children of European origin [60]. Note that, in Brazilians, it was found that the homozygous mutant genotype GG increases the risk of ALL 17-fold [81], in concordance with the findings described in Mexican children [66]. However, this polymorphism showed no association with ALL in the Iranian population [65].

MiR-612. The rs12803915G/A SNP is located in pre-miR-612. Functional in vitro analyses in different cancer types, such as prostate, colon, and breast, have shown that its effect in mature miR-612 expression might be dependent on the cellular and tissue-specific context [82]. It has been reported as associated with protection against ALL development in Caucasians from Spain, but studies in the Iranian population showed no association [83]. Furthermore, members of the Ikaros family of zinc-finger (IKZF) proteins have previously been associated with ALL susceptibility, and since IKZF2 transcription factor, one of the potential targets of miR-612, is involved in the regulation of lymphocyte development, the effect of the mutant allele rs12803915 A in the regulation of miR-612 could explain an association with ALL risk, but studies in different cohort of patients might be performed to confirm such results [60,84,85].

MiR-34b/c. Associations between the miR-34b/c rs4938723T/C polymorphism (located within the CpG island of the pri-miR-34b/c promoter region) with cancer risk have been studied extensively [86,87,88,89,90], but its influence in ALL was documented till 2016 by Tong et. al. These authors reported that the rs4938723 CC genotype was associated with a reduced ALL risk in Chinese children and showed that the transcription activity of miR-34b/c was increased when T allele transited to C through in vitro luciferase assays in Jurkat and K-562 cell lines [91]. In addition, Hashemi et. al. reported that the C allele significantly decreased the risk of childhood ALL compared to the T allele in the Iranian population [92].

Other miRNAs. MiR-100 (rs543412C/T) and miR-938 (rs2505901T/C) polymorphisms have been explored lately in ALL. Subjects carrying mutant homozygous TT genotype of miR-100 rs543412C/T had a statistically significantly decreased risk of childhood ALL in the Chinese population; in this same study, it was also documented that miR-100 expression was lower in controls than in cases. In addition, it was found that individuals with TT genotype had a significantly lower level of miR-100 compared with the wild CC genotype [68]. Moreover, rs2505901C/T, located in miR-938 and described as a gene responsible for the regulatory pathways of the genes related with cell survival and apoptosis, showed an association between the wild-type homozygous CC genotype and a lower risk of childhood ALL development in a population in Brazil [81].

Genes involved in miRNAs’ processing/maturation. The genetic variants located in genes involved in miRNA processing and maturation may also affect levels of miRNA expression. For instance, rs10035440 in *DROSHA*, and rs9606248 and rs1640299 in *DGCR8*, which have been associated with ALL risk, could modify miRNA by altering *DROSHA* and *DGCR8* expression levels [60]. Recently, the genotype AA of the SNP rs3805500 (*DROSHA*) was documented to be associated with an increased risk of developing ALL when compared to other genotypes. This variant is in linkage disequilibrium with rs640831 (*DROSHA*), which has been related to an alteration in the maturation of pri-miRNAs and pre-miRNAs [81]. The knowledge about the role of SNPs located in genes involved in miRNAs’ biogenesis is still scarce; thus, future research directions should be focused on this topic.

### 4.2. MiRNAs’ SNPs and Drug Metabolism

There is no doubt about the relevance of SNPs as modifiers of drug metabolism and toxicity [93,94,95]. A study of SNPs located in miR-5189, miR-595, and miR-6083 genes reported that the rs56292801AA genotype of miR-5189 is associated with protection against methotrexate (MTX) accumulation and toxicity over time. In silico studies revealed that *SLC46A1* is the target gene predicted for this miRNA, which encodes for the proton-coupled folate transporter, involved in MTX transport [96,97]. Likewise, the rs4909237TT genotype in miR-595 showed an increased risk of having MTX high plasma levels over time. In addition to *SLC46A1*, miR-595 target genes include the transporters *SLC19A1* and *SLCO1A2*, which are also involved in MTX uptake [98]. Another miRNA gene associated with MTX concentration is miR-6083, and its rs78790512GG and AG genotypes have been associated with the accumulation of MTX over time. Since no mRNA has been identified as a miR-6083 target, it is speculated that it could have an indirect effect by regulating other ncRNAs [99]. As miRNAs can also regulate genes involved in drug transport, metabolism, and targets, SNPs in miRNA biogenesis proteins are not only related to a predisposition for pediatric ALL but may also participate in drug response or resistance, such as rs639174 in *DROSHA*, an intronic SNP that has been associated with toxicity during chemotherapeutic treatment [100,101]. Notably, based on the HaploReg v4.1 online tool for variant annotations [102], the rs639174 (*DROSHA*) variant could modify the binding sites for glucocorticoid receptor (GR) sequence. GR is a critical receptor in ALL, since glucocorticoid (GC) plays an essential role during the treatment of ALL, and mutations in this gene are associated with GC resistance [103].

### 4.3. MiRNAs’ Expression Profiles and Their Role as Biomarkers in ALL

Several studies have documented that the miRNAs’ expression profiles in ALL could be useful to distinguish between normal and pathological hematopoiesis (diagnostic biomarkers), leukemia lineages, molecular subtypes, relapse, and prognostic risk prediction [104,105,106,107].

#### 4.3.1. Diagnostic Biomarkers

One of the first studies aimed at identifying miRNAs differentially expressed between ALL and normal samples was performed by Zanette et al. in 2007. The authors assessed the expression of 164 miRNAs in CD19+ B cells from healthy individuals and ALL patients, identifying 45 miRNAs differentially expressed between both groups. The most overexpressed were miR-128b, miR-204, miR-218, miR-331, and miR-181b, and the most underexpressed were and miR-135b, miR-132, miR-199, miR-139, and miR-150 [108]. Furthermore, a study comparing CD34+ B cells from ALL patients and healthy subjects reported 15 miRNAs differentially expressed among groups. In that study, the most upregulated miRNAs were miR-128a, miR-142, miR-150, miR-181, miR-30e-5p, miR-193, miR-34b, miR-365, miR-582, and miR-708, and the highest downregulated miRNAs were miR-100, miR-125b, miR-99a, miR-196b, and miR-let-7e [109]. Other studies contributed to the identification of miRNAs abnormally expressed in ALL cases versus normal samples observing a higher expression of many other miRNAs, as described in Table 1. Nevertheless, only miR-125b-1, miR-128, miR-128(a-c), miR-142-p3, miR-155, miR-181a, miR-181b, miR-181b-5p, and miR-203 are among the most consistently replicated in ALL samples at the time of diagnosis [107,110,111,112,113,114,115,116,117]. Recently, it was reported that miRNAs enriched in exosomes and vesicles of circulating leukemic cells, such as miR-181b-5p, could also be useful as a prognosis biomarker for child ALL [115]. It has been demonstrated that miR-181b-5p contributes to the proliferation, migration, and invasion of leukemic cells [115]. By using small RNA sequencing, some differentially expressed miRNAs have also been found in T-lineage ALL compared to healthy bone marrow, with miR-548a-3p, miR-128-3p, miR-181b-5p, miR-20b-5p, miR-574-5p, miR-10a-5p, miR-582-3p, and miR-143-3p being among the most deregulated [110,118].

At this time, many efforts have been made to improve the detection of hematological malignancies by using specific miRNA that could perform better than other markers for disease diagnosis; however, few of them have been validated in more than one study, and none of them has reported a consensus miRNA signature, revealing the complexity of the biology of ALL and the potential involvement of technical and sample-size biases during the studies performed.

#### 4.3.2. Immunophenotype Classification

The identification of miRNAs as potential biomarkers for subtyping linages in ALL has been a constant objective in diverse studies, but the results are still controversial [48,49,51,109,110,126,127]. 

For instance, several miRNAs were found to be abnormally expressed in T-cell ALL in contrast to B-ALL, as shown in Table 2, but few of them have been replicated in at least two different reports like miR-151, miR-424, miR-29c-5p, and miR-708 have [126,127,128,129]. Moreover, miR-29c-5p has been considered to be the best discriminator between childhood T- and B-ALL, due not only to its upregulation in T-ALL, but also because among the potential targeted genes of this miRNA are some participating in ALL development, such as *AFF1, KMT2A*, and *ELK4* [126].

#### 4.3.3. Molecular Subtype Identification

The impact of miRNAs on ALL molecular subtype classification is one of the mayor fields to which miRNA expression profiles have contributed. For instance, Schotte et al., when evaluating 397 miRNAs, found that these genes cluster in specific expression patterns, which are useful to differentiate among mixed-lineage leukemia (*MLL*)-rearrangements, *TEL-AML1*-positive, *E2A-PBX1*-positive, and hyperdiploidies, but not *BCR-ABL1*-positive ALL and “B-other” ALL (lacking recurrent fusion genes) [110]. In addition, a study focused on ALL patients with microdeletions (mainly in *IKZF1*), and it was demonstrated that the expression level of miR-128 was significantly lower in this group of patients than it was in their counterparts. In addition, cases with *CDKN2A/B* and miR-31deletions had a low expression of miR-542, and patients who were positive to miR-31 and *PAX5* deletion displayed low expression of miR-24, miR-708, and miR-128; meanwhile, high expression of miR-24 and miR-542 was associated with *PAR1* deletion, and overexpression of miR-708 was associated with *ETV6* deletion [131].

*MLL*-rearranged leukemias are a group of aggressive leukemias that is often developed in infants. Recently, upregulated miR-130b and miR-128a were identified in *MLL-AF4* primary samples [132]. Interestingly, miR-130b and miR-128a recapitulated *MLL-AF4* leukemias with unique lineages in murine models, underlining the complexity of the mechanisms that drive leukemogenesis [132]. The loss of expression of other miRNAs, such as miR-432, miR-503, and miR-148a, which are markedly downregulated in *MLL*-rearranged leukemia, is predicted to target and overdrive the expression of several genes associated with early recurrence and poor outcome [133]. The knowledge of miRNAs in normal and pathological hematopoiesis has been obtained from murine models and cell lines. For instance, studies in murine models positive to *MLL-AF4* fusion protein encoded by the chromosomal translocation t(4;11) show that miR-128a and miR-130b can drive the transition from a pre-leukemic to acute leukemia stage and that both miRNAs are needed to maintain the leukemic phenotype. An interesting fact was that miR-130b resulted in a mixed/B-cell precursor (BCP)/myeloid leukemia, while miR-128a was conducted to a pro-B ALL [132]. In cell lines carrying t (4;11), it was observed that miR-142-3p inhibits the *MLL-AF4* expression [134].

Other subtypes recently described, such as *ERG*-altered ALL, have also been associated with miRNAs’ expression signatures, with the upregulation of the known miR-125b-2 cluster being characteristic of this subtype [135]. In another novel subtype that involves gene fusions with the myocyte enhancer factor 2 gene (called *MEF2D*-rearranged), it was shown that the miR-122 is a critical repressor of *MEF2D*. The loss of the miR-122 target site by chromosomal translocation leads to *MEF2D* overexpression, acting cooperatively with the transcriptional activities of *MEF2D* that include *PAX5* inhibition and the arrest of B cells’ differentiation [136].

#### 4.3.4. Disease-Free Survival and Overall Survival

Deregulated miRNAs’ expression has been explored as a diagnostic biomarker associated with disease-free survival (DFS) and overall survival (OS) to identify patients with a high risk of relapse or detect the minimal residual disease (MRD).

Among the miRNAs associated with unfavorable long-term clinical outcome are miRNA-21, miR-33, miR-143, miR-182, miR-215, miR-369-5p, miR-496, miR-518d, miR-599, miRNA-155a, and miR-181a [137,138]. In addition to a poor prognosis, miRNA-21 upregulation has been associated with high-risk clinical features, such as age, lower platelet count, and worse disease-free survival and OS [139]. Moreover, miR-125b, which has a critical role in B-cell development, has been suggested as an independent prognostic factor. The downregulation of this ncRNA correlates with the inferior outcome at diagnosis, but on the other hand, its high expression on day 33 (after induction phase) was associated with short-term survival and worse OS [140]. To note, high levels of miR-143 and miR-182 at the end of induction therapy were associated with a higher risk of short-term relapse and death (Figure 2B) [141]. 

Overall, miR-10a, miR-134, miR-214, miR-484, miR-572, miR-580, miR-624, and miR-627 have been linked to a favorable outcome, and the first three (miR-10a, miR-134, and miR-214) were previously reported as tumor suppressors in other types of cancers. Moreover, miR-10a and miR-214 work by inhibiting cell proliferation, and miR-134 works by downregulating the *SOX2* (sex-determining region Y-box 2) transcription factor that has been identified as an oncogene [110,142,143,144]. Additionally, miR-23a, miR-27a, miR-128b, miR-181, and miR-223, showed a differential expression pattern when comparing samples obtained at complete remission versus diagnostic times. The expression profile was also significantly distinct among the relapse and non-relapsed groups. Since miR-223 has previously been identified as a tumor-suppressor gene and an important factor in leukemogenesis, this can explain its overexpression in complete remission patients [137,145].

Relapse. Both miR-922 and miR-1324 were differentially expressed miRNAs associated with early relapse (relapses that occur within 36 months from initial diagnosis) [146]. These miRNAs were previously identified in other types of cancer but not in ALL. MiR-708, miR-27a, and miR-223 have been proposed as potential biomarkers due to the correlation of a higher relapse free survival rate with high expression of these three miRNAs in ALL pediatric patients. Moreover, their target genes are implicated in leukemic cell development, differentiation, and activation [137].

Another study showed that a gene-expression signature comprising a low expression of miR-151-5p and miR-451 and the high expression of miR-1290 is an independent prognostic biomarker of relapse in B-ALL (10.5-fold increased risk). This signature maintained a significant association with relapse, even when excluding patients with unfavorable cytogenetic markers, and *IKZF1* deletion (24.5-fold increased risk) [147]. Han et al. showed a miRNA expression profile in pediatric ALL patients in complete remission that was significantly distinct from that of patients that presented relapse. MiR-223, miR-23a, and let-7g were downregulated, whereas miR-181 family, miR-708, and miR-130b were upregulated in the relapse samples [137]. Moreover, a significant association was observed between miR-24 overexpression and the risk of relapse, as well as a shorter overall survival compared to those with low miR-24 expression [148].

Minimal Residual Disease. In addition, to discriminate between ALL patients versus healthy controls, it was reported that miRNA-155a is a potential biomarker to evaluate MRD [138]. Recently, Rzepiel et al. (2019) reported that circulating miR-128-3p and miR-222-3p in blood predict day 15 flow cytometry MRD results 7 days earlier; thus, these miRNAs may act as biomarkers of residual leukemia [149].

Chemotherapeutic Response and miRNA Expression. The survival rate in ALL has been improved over the last two decades; however, the mortality proportion due to toxicity of chemotherapy still happens. Thus, it is relevant to identify biomarkers that can help to detect those cases that are at a high risk of developing toxicity, especially to the cornerstone of chemotherapy agents (vincristine, asparaginase, daunorubicin, glucocorticoids, cyclophosphamide, cytarabine, methotrexate, and the thiopurines mercaptopurine and thioguanine) [150]. For example, Zhang et al. identified a unique miRNA-expression signature composed of eight miRNAs (miR-18a, miR-532, miR-218, miR-625, miR-193a, miR-638, miR-550, and miR-633) that can differentiate between a good or poor prednisone response in pediatric ALL [151].

In ALL cell lines and prednisone-resistant patients’ samples, it was found that miR-124 and miR-331-3p were upregulated, potentially by repressing the glucocorticoids (GC) receptor expression and by the inhibition of the JNK/MAPK pathway [152,153]. By conducting a microarray analysis of ALL cases, it was found that miR-185-5p is overexpressed; furthermore, it was observed that its overexpression increases cell apoptosis and cycle arrest and decreases cell survival GC resistance cell line by targeting one component of the mammalian target of rapamycin complex, a pathway involved in several types of cancer and associated with GC resistance in hematological malignancies [154].

To identify if miRNA is associated with prednisolone, vincristine, L-asparaginase, and daunorubicin resistance, Schotte et al. studied 81 ALL cases and found that sixteen miRNAs were discriminative for resistance to one or more drugs. MiR-454 has been expressed at a 1.9-fold-lower level in L-asparaginase-resistant cases in comparison to their counterparts, whereas miR-125b, miR-99a, and miR-100 were the most upregulated in patients resistant to vincristine and daunorubicin [110]. Otherwise, experimental studies revealed that the co-expression of miR-125b and miR-99a or miR-100 increases the cellular viability of REH cell lines treated with vincristine [155].

Han et al. found that miR-708 was significantly upregulated in prednisone good-response patients, indicating that the miR-708 level before chemotherapy could be highly predictive of prednisone response [137]. MiR-125b has been also reported as being associated with chemotherapy resistance in B-cell malignant multiple myeloma, for which the expression can prevent cell death through suppression of p53 and the p53/miR-34a/SIRT regulatory network [140,156]. 

In addition, specific miRNAs have been studied in vitro to better understand their participation and function in drug response; for example, the relevance of the level of expression of miR-210 was studied by Mei et al. Their results outlined that increasing/decreasing miR-210 expression could enhance or reduce the response of leukemic cell lines to daunorubicin–dexamethasone–L-asparaginase and daunorubicin–dexamethasone–vincristine, respectively. These findings suggest that an increased intracellular level of miR-210 enhances the sensitivity of leukemic cells to common chemotherapeutic drugs and decreases their viability [157].

Another relevant miRNA is miR-652-3p because it has been documented that increased levels of its expression might assist in suppressing lymphoblastic leukemia cells. Jiang et. al. suggest that the expression of miR-652-3p is markedly lower in the lymphoblastic leukemia cell lines compared with the normal B-cell lines and implied that the increase in apoptosis induced by the overexpression of miR652-3p might, to some extent, contribute to the increased sensitivity of lymphoblastic leukemia cells to chemotherapeutic drugs, particularly vincristine and cytarabine [158].

In addition to the in vitro and in vivo models and ALL samples’ findings, computational tools have also been useful to predict miRNAs’ deregulation and their implications for prognosis and therapy resistance. Regarding this, a semantics-based search approach predicted that miR-142-3p and miR-17-5p potentially regulate GC resistance, being some of the most promising and validated markers across different studies found in the literature [159].

ALL cells co-cultured with bone-marrow stromal cells or primary human osteoblasts lead to the downregulation of miR-221 and miR-222 and G0 cell-cycle arrest; conversely, induced expression of miR-221 sensitized leukemic cells to chemotherapy agents and allowed cell-cycle progression [160]. The crucial role of the bone-marrow niche for both HSCs and leukemic cells is known. The bone-marrow niche provides cues that ensure survival and quiescence of HSCs, a context that leukemic cells with stem-like features benefit from, often leading to resistance to therapy and relapse.

There are encouraging findings in miRNA research for pediatric leukemias. Despite concerns that sncRNAs’ stability could limit their accurate detection, there is a high correlation between miRNAs’ expression in body fluids, such as plasma, serum, urine, bile, and feces, and their tissues of origin, highlighting their potential benefit as clinical biomarkers for disease-specific monitoring [161,162,163]. Comprehensive tools are still needed to find the most accurate miRNAs for prognostic purposes in ALL since accuracy and reproducibility are still important caveats in this research area.

## 5. Molecular Mechanisms of miRNAs in ALL

The most straightforward approach of miRNA functional mechanisms is through mice models, cell-lines studies, and functional enrichment analysis (FEA) using the miRNA-target genes. FEA use databases that have been developed to infer miRNA functions and identify targeted genes (miRGator, miRDB, miRò, MAGIA and FAME, miRanda, PicTar, and TargetScanS); thus, it can give us a large amount of knowledge of the potential biological role of them [164]. Nevertheless, few studies have been conducted to increase our understanding of the molecular mechanisms of miRNAs underlying ALL development [155].

Among the most replicated miRNAs, miR-125b is considered to be a diagnostic and prognostic biomarker, and it is a key miRNA that is implicated in T-cell maturation. This ncRNA is overexpressed in undifferentiated T-cells and induces glucose metabolic switch in T cells via targeting TNF-α-induced protein 3, which is frequently found to be inactivated, mutated, or deleted in leukemia. It also increases cell growth and in vivo invasiveness, contributing to T-ALL development [48,49]. Furthermore, miR-125b acts as a critical tumor suppressor since its abnormal expression impairs the exit of immature B cells from the bone marrow, and its silencing is necessary for normal B-cell development [46].

Moreover, miR-128 has been reported to show dysregulation in several tumors that results in inhibition or promotion of tumor proliferation. The transcriptional factor and oncogene proto-oncogene polycomb ring finger, which is important in hematopoietic stem cells and leukemia stem-cell self-renewal, has been predicted to be one of its putative targets, and in silico predictions suggest that it is one of the major regulators of lymphoid differentiation. It was proposed as a potential biomarker to discriminate between AML and ALL, as well as to predict MRD [107,120,149,165].

Experimental studies have suggested that miR-26b, miR-20b-5p, and hsa-miR-363-3p could be relevant to T-ALL development [128,166]. For instance, miR-101, which is frequently downregulated in primary T-ALL samples, plays a tumor-suppressor role, as it suppresses the expression of Notch1 and the proto-oncogene *TAL1* [167,168].

When comparing with normal marrow cells, miR-196b was found to be upregulated in ALL patients positive to *MLL*-rearrangements and in those with T-ALL. Evidence shows that the presence of *MLL* fusion proteins leads to aberrant mir-196b expression, causing increased proliferation and abnormal hematopoietic differentiation and contributing to leukemogenesis. *MLL* regulates the expression of the homeobox domain (*HOX*) gene family that is involved in the regulation of normal hematopoiesis. Aberrant expression of *HOXA* genes is not restricted to *MLL*-rearranged precursor B-ALL cases; it has also been reported in T-ALL patients. MiR-196b is mapped between the *HOXA9* and *HOXA10* genes on chromosome 7p15.2, so the level of expression of miR-196b may be linked to *HOXA* gene transcription [169,170].

A bioinformatic analysis of public gene-expression datasets revealed that miR-124-1, miR-124-2, and miR-124-3 are overexpressed in ALL patients with early recurrence. Interestingly, it was predicted that the tumor-suppressor gene, interferon-induced protein-44-like gene is targeted by miR-124-3p, which displays the potential therapeutic implications of this ncRNA [171].

## 6. Closing Remarks and Conclusions

It has been widely documented that miRNAs’ deregulation in cancer is often due to structural alterations (chromosomal translocations, deletions, and amplifications) in regions that contain them, or perturb genes that control their expression. Particularly in hematological malignancies, there are some examples, such as loss of 13q14 region in CLL, that contain miR-15 and miR-16, which are important regulators of BCL2-mediated apoptosis, leading to a reduced expression or loss of expression [172,173]; the proto-oncogene *MYC*, frequently altered by chromosomal rearrangements and amplifications, regulates several miRNAs, such as the miR-17-92 cluster, miR-34a, and miR-15a/16-1, and its expression is also controlled by miR-17-92 in a feedback-loop manner [174]. Furthermore, miRNAs are involved in most aspects of ALL biology, observed as differential expression profiles associated with the disease, molecular subtypes, prognosis, and drug response. Although the studies reveal a great heterogeneity on the miRNAs involved in any clinical aspect of ALL, the findings that miR-125 and miR-142 have been detected in several studies point to the fact that these miRNAs have potential for applications in the clinical field. Proper validation to ensure reproducibility across different technologies and methodologies is key for their implementation into the clinical setting and for patients’ benefit.

Overall, miRNAs’ therapeutic potential is encouraging but still faces major challenges. The fact that miRNAs can regulate numerous cancer-related pathways makes them attractive therapeutic targets, and many technical approaches are currently evaluated for the purpose of restoring miRNAs’ function in diverse diseases, namely as miRNA sponges, antisense antagomers, small molecule inhibitors, miRNA editing, and non-viral delivery systems [175,176,177,178]. Unfortunately, only a few efforts have progressed into clinical trials, and miRNAs have been mostly proposed as diagnosis or prognosis markers. The improvement of delivery systems could enhance their therapeutic application by diminishing the off-target effects seen in preclinical studies and by increasing their stability for tissue or disease-specific activity. 

Thus, the knowledge stated so far here and the continuous interest of many research groups in understanding the biological effect of miRNAs expression are of great relevance not only because they could be implemented as non-invasive diagnostic biomarkers, but they could also help in regard to ALL diagnosis, risk stratification, and relapse and toxicity prediction. This could be translated into a better personalized therapy for specific groups of patients that share specific expression miRNA profiles, which could help to improve survival and reduce the risk of secondary adverse effects.

## Figures and Tables

**Table 1:** Abnormally expressed miRNAs in acute lymphoblastic leukemia patients compared to healthy individuals.

Upregulated	Downregulated
miR-7e [111], miR-9 [111], miR-9* [111], miR-34a [116], miR-92a [113], miR-100 [119], miR-125b-1 [114], **miR-128** [111,120,121], miR-130b [111], miR-142-3p [112], **miR-146a** [115,119,122], miR-155 [115], miR-181 [121], **miR-181a** [111,117], **miR-181b** [111,115], miR-210 [123], **miR-222** [112,116], miR-339 [112], miR-363 [111], miR-511 [116], miR-638 [113], miR-1943 [111], miR-1841 [111], miR-1931 [111], miR-1987 [111], miR-1890 [111], miR-1902 [111]	let-7e [121], miR-18a [124], miR-26a [116], miR-30a [111], **miR-100** [121,123], miR-126 [111], miR-143 [111], miR-145 [115], miR-196a [119], **miR-196b** [117,121], miR-199b-3p [111], miR-200c [125], miR-203 [114], miR-221 [116], **miR-223** [111,116], miR-326 [125], miR-373* [112], miR-451 [112], miR-582-5p [111], miR-1893 [111], miR-1971* [111], miR-1834 [111], miR-1842* [111], miR-1842 [111]

MiRNAs in **bold** letters are described in more than one paper.

**Table 2:** The miRNAs that are differentially expressed in T-ALL compared with B-ALL.

Upregulated	Downregulated
miR-29c-5p [126], miR-137 [129], miR-148a [127], miR-149* [130], miR-193b* [129], miR-424-5p [126], miR-424 [127], miR-450b-5p [126], miR-450a-5p [126], miR-506 [129], miR-509-5p [129], miR-510 [129], miR-542-5p [126], miR-629-5p [126]	miR-100 [128], miR-151 [127], miR-151a-5p [126], miR-151b [126], miR-195-5p [126], miR-371b-5p [126], miR-383 [129], miR-425-5p [126], miR-455-5p [126], miR-497-5p [126], miR-574-5p [126], **miR-708** [128,129], miR-708-5p [126], miR-1266-5p [126]

MiRNAs in **bold** letters are described in more than one paper.

## Data Availability

Not applicable.
